# Impact of prehospital antibiotic therapy differs depending on septic shock origin

**DOI:** 10.1186/s40560-026-00853-y

**Published:** 2026-03-02

**Authors:** Romain Jouffroy, Vincent Garrouste, Basile Gilbert, Stéphane Travers, Daniel Jost, Emmanuel Bloch-Laine, Patrick Ecollan, Vincent Bounes, Josiane Boularan, Benoit Vivien, Papa Gueye

**Affiliations:** 1 LI2RSO, Orléans University Hospital Orléans University, 45100 Orleans, France; 2 Institut de Recherche bioMédicale Et d’Epidémiologie du Sport - EA7329, INSEP, Paris University, Paris, France; 3 Centre de Recherche en Epidémiologie Et Santé Des Populations - U1018 INSERM, Paris Saclay University, Paris, France; 4 Service de Médecine du Sport, Orléans University Hospital, 45100 Orleans, France; 5 LI2RSO, Orléans University Hospital, Orléans University, 45100 Orléans, France; 6 Department of Emergency Medicine, SAMU 31, University Hospital of Toulouse, Toulouse, France; 7 Paris Fire Brigade, French Military Health Service, Paris, France; 8 Emergency Department, Cochin Hospital, Paris, France; 9 Emergency Department, SMUR, Hôtel Dieu Hospital, Paris, France; 10 Intensive Care Unit, SMUR, Pitie Salpêtriere Hospital, 47 Boulevard de L’Hôpital, 75013 Paris, France; 11 SAMU 31, Castres Hospital, Castres, France; 12 SAMU 972 University Hospital of Martinique (CHU de Martinique), Fort-de-France, Martinique France; 13 Cardiovascular Research Team (UR5_3 PC2E), University of the French West Indies, (Université Des Antilles), Fort de France, Martinique France

**Keywords:** Septic shock, Antibiotic therapy, Origin, Prehospital, Mortality

## Abstract

**Background:**

International guidelines recommend early a bundle of care to reduce sepsis mortality. Among bundle of care, antibiotic therapy is all the additionally effective when early initiated, especially for the sicker patients, i.e., those with septic shock, for whom it should be started within the first hour. This study aims to examine the impact of prehospital antibiotics administration on 30-day mortality in patients with septic shock, as defined by Sepsis-2, cared for by a prehospital mobile intensive care unit (MICU).

**Methods:**

We performed a nationwide observational cohort study in France using data from May 2016 to December 2021 including septic shock patients admitted to ICU after receiving prehospital care from a MICU. An emulate retrospective randomized controlled trial using a weighted Cox proportional hazards model was conducted to compare the efficacy of prehospital antibiotic administration versus no prehospital antibiotic administration on 30-day mortality. A secondary analysis assessed the association between prehospital antibiotic administration and 30-day mortality according to presumed septic shock origin.

**Results:**

Among the 530 patients analyzed, 341 (64%) were males and the mean age was 70 ± 15 years. The 30-day mortality was 31%. The presumed origins of sepsis in the prehospital setting were primarily pulmonary, digestive, and urinary, with respective percentages of 43%, 25%, and 17%, respectively. One-hundred and thirty-two patients (25%) received prehospital antibiotic therapy, a 3rd generation cephalosporin for 98 patients (18%). The inverse probability of treatment weighting analysis emulating the target trial revealed that prehospital antibiotic administration was associated with a lower risk of 30-day mortality compared with no prehospital antibiotic administration: RR = 0.64, 95%CI [0.41–0.97]. The weighted logistic regression model showed a significant association between 30-day mortality and prehospital antibiotic administration for pulmonary origin: RRa = 0.80 [0.86–0.93], urinary origin: RRa = 0.89 [0.80–0.98], and unknown origin: RRa = 0.94 [0.86–0.99].

**Conclusion:**

The prehospital antibiotics administration is associated with a reduced risk of 30-day mortality among patients suffering from septic shock cared for by a prehospital MICU. The prehospital antibiotic treatment effect differs according to septic shock origin. However, prospective studies are necessary to validate these preliminary findings and to assess the supplementary effects of the bundle of care components.

## Introduction

Sepsis is a life-threatening disease responsible for substantial mortality and morbidity [1–4]. Overall mean sepsis-related mortality rate is close to 25–30% but can reach 50 to 60% for septic shock [5–7]. International consensus conferences recommend a bundle of care consisting of sepsis early recognition, severity assessment, and treatments especially for the sicker patients, i.e., for septic shock [4, 8]. Among treatments, antibiotic is more effective when early initiated, especially for septic shock. Consequently, to date, for these latter’s patients, a “1-h bundle of care” starting within the first hour after sepsis recognition is, to date, standard of care [8, 9]. Within the bundle of care, the surviving sepsis campaign guidelines [3, 8, 10] recommend early antibiotic administration in association, when necessary, i.e., surgery for peritonitis, with source control to improve outcome [8, 10–12]. Recently, Lee et al. reported a positive association between the prompt administration of appropriate antimicrobials and a favorable prognosis, particularly among critically ill patients, regardless of the need for source control for treatment-naïve adults admitted to the emergency department with bacteremia [13].

Nearly, 70% of sepsis occur outside hospital environments [14] with a median time to reach hospital is around 1 h [15]. We previously reported that a prehospital antibiotic administration among septic shock patients cared for by a prehospital mobile intensive care unit (MICU) was safe and associated with 30-day mortality decrease [15, 16]. However, the antibiotic therapy effect alone is also related to sepsis origin.

This study aims (i) to assess the impact of prehospital antibiotics administration on 30-day mortality in patients with septic shock cared for by a prehospital MICU and (ii) to investigate its association according to presumed origin of septic shock.

## Methods

### Study setting

French prehospital emergencies are managed by the “SAMU” (Urgent Medical Aid Service). “SAMU” is a public health control organization with a central dispatch center that may dispatch a prehospital MICU, named SMUR in French (Mobile Emergency and Resuscitation Service), to the scene to provide medical assistance when necessary [14]. MICU is composed by a driver, a nurse, and an emergency physician and equipped with medical devices and drugs to support respiratory, hemodynamic, and neurological failures [17].

### Design

We performed a nationwide observational cohort study in France using prospectively collected data from May 2016 to December 2021. Patients cared for by one of the 9 prehospital MICU French hospital centers (Necker-Enfants malades Hospital, Lariboisière Hospital, La Pitié Salpêtrière Hospital, Hotel Dieu Hospital, APHP, Paris – France; Paris Fire Brigade, Paris – France; Toulouse University Health Centre, Toulouse – France, Martinique University Health Centre, Fort de France – France, and the Castres Hospital, Castres – France) were analyzed. According to the 2012 Sepsis-2 Conference criteria [19], septic shock was diagnosed in the prehospital setting by the MICU physician and followed during 30 days.

The data were collected from hospital medical records of adult patients suffering from septic shock and admitted to the ICU after receiving prehospital care from a MICU. All patients who received MICU care in the prehospital setting were subsequently admitted to the ICU after hospital arrival.

### Patients

Adult patients diagnosed with septic shock in the prehospital setting by the MICU physician were included in the study. Patients younger than 18 years, pregnant, with guardianship or curatorship were not included in the dataset [18]. We choose the Sepsis-2 definition considering a septic shock a condition of refractory hypotension despite vascular filling or normotension with hypoperfusion signs. This decision was made in light of the unavailability of blood tests in the prehospital setting and the limited availability of lactate level assessments in all MICU. [21]. Consequently, the Sepsis-2 definition emerges as a more suitable and practical approach when applied in the prehospital context compared to the Sepsis-3 definition.

To date, in France, there are no guidelines for prehospital sepsis management. Hemodynamic support, i.e., fluid expansion and/or catecholamine infusion, as well as antibiotic administration, are left to the MICU physician discretion. Available antibiotics in French prehospital MICU are 3rd generation cephalosporin (cefotaxime and ceftriaxone), amoxicillin/clavulanate, and piperacillin/tazobactam; antibiotic therapy choice and dose are left to the MICU physician discretion. To date, in France, no blood culture is performed because of the absence of the French MICU capacity.

In France still, a retrospective study reported that nearly 30% of septic shock cared for in the prehospital setting by a MICU benefit from antibiotic administration [19].

### Data collection

Patients’ demographic characteristics (age, weight, height, and gender), prehospital presumed clinical origin of sepsis (e.g., on the emergency scene), initial prehospital (e.g., the first MICU contact), and final prehospital (e.g., at the end of prehospital stage) vital sign values (systolic (SAP), diastolic (DAP), and mean arterial pressure (MAP)) were measured with a non-invasive automated device in all centers. Heart rate (HR), pulse oximetry (SpO2), respiratory rate (RR), body core temperature and Glasgow coma scale (GCS)), plasma blood glucose concentration, duration of prehospital care (duration between MICU arrival on scene and hospital admission), and prehospital treatments delivered (antibiotic therapy type and dose, fluid volume expansion type and dose, as well as norepinephrine with dose expressed as the base equivalent) were collected from MICU prehospital medical reports. Major comorbidities, i.e., hypertension, coronary heart disease (CHD), chronic cardiac failure (CCF), chronic renal failure (CRF), chronic obstructive pulmonary disease (COPD), diabetes mellitus, and history of cancer were also collected from MICU prehospital and hospital medical reports to take into account the underlying condition [20]. Patients referred from other medical facilities were not included in the scope of this study.

Length of stay (LOS) in the ICU, in-hospital LOS, and 30-day mortality were retrieved from medical reports in case of in-hospital death or by call when the patient was discharged from the hospital. Sequential Organ Failure Assessment (SOFA) score [21] was calculated at hospital admission and simplified acute physiology score (SAPS-2) [22] was calculated 24 h after ICU admission.

According to article R.1123–46 of the *Code de la Santé Publique* (French Public Health Code) definition, adverse reaction, i.e., any adverse event occurring in a person enrolled in a study involving human participants, when this event is related to the study or to the product being studied, was documented in the database.

A standardized abstraction template established prior to the study and a data check identifying no error were used to minimize data abstraction bias [23].

As previously reported [19], this study was approved by the French Society of Anaesthesia and Intensive Care ethics committee on December 12th, 2017 (Ref number: IRB 00010254–2017-026). According to the French law, in this non-interventional observational study the ethical committee waived consent of patients.

### Data analysis

Quantitative parameters with a Gaussian distribution are expressed by mean with standard deviation, quantitative parameters with a non-normal distribution by median with interquartile range [Q1–Q3], and qualitative parameters by absolute value and percentage.

First, 30-day mortality rate was reported among patients with prehospital antibiotic therapy vs. patients without prehospital antibiotic therapy depending on prehospital suspected septic shock origin.

Second, a target trial analysis with Inverse Probability of Treatment Weighting (IPTW) method was performed to take into account for potential confounders. The target trial emulated by this observational analysis was defined as follows:

- The eligible population included adult patients presenting with septic shock according to the Sepsis-2 definition and managed in the prehospital setting by a prehospital MICU.
- Patients younger than 18 years, pregnant women, and patients under legal guardianship or curatorship were excluded.
- Time zero was defined as the initiation of prehospital care by the SMUR team.
- Patients were classified according to the treatment strategy initiated at time zero: [1] prehospital antibiotic administration or (2) no prehospital antibiotic administration assigned at time zero.
- Treatment assignment was not randomized.
- The main outcome was all-cause mortality at 30 days.
- All patients were followed for 30 days from time zero with follow-up starting at the same time, i.e., on the day of prehospital care initiation to avoid immortal time bias.
- All patients were analyzed according to their initial treatment strategy, regardless of subsequent in-hospital antibiotic management.
- The baseline covariates included were age, SOFA, CRF, COPD, CHD, history of cancer, prehospital norepinephrine dose, prehospital lactatemia, body mass index, diabetes mellitus, initial mean arterial pressure, initial RR, initial pulse oximetry, initial HR, initial GCS value, and prehospital fluid volume expansion.
- The causal estimand was the intention-to-treat (ITT) effect of prehospital antibiotic administration on 30-day mortality.
- To account for confounding, propensity scores representing the probability of receiving prehospital antibiotics conditional on baseline covariates using logistic regression was used. IPTW based on the propensity score was applied to create a weighted pseudo-population in which baseline covariates were balanced between treatment groups. Balance between groups was assessed using standardized mean differences.
- The primary analysis, the effect of prehospital antibiotic administration on 30-day mortality, was estimated using a weighted Cox proportional hazards model, and the results are expressed as risk ratio (RR) with 95% confidence intervals (CI). Proportional hazards assumptions were assessed using standard diagnostic methods.
- A secondary analysis with weighted logistic regression model including the same potential confounders, assessed the association between prehospital antibiotic administration and 30-day mortality according to presumed septic shock origin. The results are expressed as adjusted RR (RRa) with 95% CI.

Missing data in baseline covariates were handled using multiple imputation prior to propensity score estimation. No formal sensitivity analyses were conducted. All analyses were performed under the assumptions of consistency, exchangeability, positivity, and correct model specification.

All tests were 2 sided with a statistically significant *p-value* < 0 0.05.

All analyses were performed using R 3.4.2^©^ (http://www.R-project.org; the R Foundation for Statistical Computing, Vienna, Austria).

## Results

### Patient characteristics

Among eligible patients with septic shock managed prehospital by MICU teams during the study period, a total of 530 patients were included in this analysis. Among them, 341 patients (64%) were male gender, and the mean age was 70 ± 15 years (Table 1).

**Table 1:** Population characteristics

	Overall population (n = 530)	Alive (n = 366)	Deceased (n = 164)	P-value
Age (years)	70 ± 15	68 ± 15	73 ± 14	** < 10**^**–3**^
Male gender	341 (64%)	243 (66%)	98 (60%)	0.141
Weight (kg)	74 ± 20	75 ± 20	70 ± 20	**0.014**
Height (cm)	170 ± 12	170 ± 12	169 ± 9	0.339
Initial prehospital values				
SBP (mmHg)	97 ± 30	99 ± 30	93 ± 30	0.055
DBP (mmHg)	58 ± 19	59 ± 19	55 ± 20	0.069
MAP (mmHg)	71 ± 22	72 ± 22	68 ± 22	0.064
HR (beats.min^−1^)	114 ± 28	115 ± 27	113 ± 31	0.463
RR (movements.min^−1^)	31 [22 – 36]	28 [22 – 35]	31 [25 – 38]	**0.012**
Pulse oximetry (%)	91 [85 – 96]	93 [86 – 96]	90 [83 – 95]	**0.005**
Body core temperature (°C)	38.3 [36.5 – 39.2]	38.4 [36.7 – 39.3]	38.1 [36.0 – 39.0]	**0.018**
Glycemia (mmol.l^−1^)	8.8 [6.3 – 12.0]	8.9 [6.7 – 12.3]	7.9 [5.7 – 11.2]	**0.014**
Glasgow coma scale	15 [12–15]	15 [13–15]	14 [11–15]	**0.002**
Blood lactate level (mmol.l^−1^)	5.8 ± 3.4	5.6 ± 3.2	6.3 ± 3.6	0.071
Prehospital fluid expansion (ml)	932 ± 573	944 ± 588	907 ± 542	0.523
Norepinephrine administration	155 (29%)	104 (28%)	51 (32%)	0.530
Norepinephrine dose expressed as the base equivalent (mg.h^−1^)	1.1 [0.5 – 2.0]	1.0 [0.6 – 2.0]	1.0 [1.0 – 2.0]	**0.041**
Prehospital antibiotics administration	132 (25%)	97 (27%)	35 (21%)	0.205
Prehospital duration (min)	71 ± 34	69 ± 33	74 ± 35	0.111
Prehospital suspected septic shock origin				
Pulmonary	230 (43%)	144 (39%)	86 (52%)	**0.039**
Digestive	130 (25%)	88 (24%)	42 (26%)	0.989
Urinary	88 (17%)	70 (19%)	18 (11%)	0.989
Cutaneous	33 (6%)	26 (7%)	7 (4%)	0.989
Meningeal	11 (2%)	9 (2%)	2 (1%)	0.989
Gynecological	3 (0.5%)	3 (1%)	0 (0%)	1.000
ENT	2 (0.5%)	1 (0.5%)	1 (1%)	0.988
Cardiac	2 (0.5)	2 (1%)	0 (0%)	1.000
Unknown	31 (6%)	23 (7%)	8 (5%)	0.989
Final prehospital values				
SBP (mmHg)	106 ± 25	109 ± 25	102 ± 27	0.056
DBP (mmHg)	62 ± 18	63 ± 18	60 ± 18	0.058
MAP (mmHg)	77 ± 19	78 ± 19	74 ± 20	0.064
HR (beats.min^−1^)	107 ± 25	107 ± 25	106 ± 28	0.396
RR (movements.min^−1^)	26 [19–30]	24 [17–30]	26 [20 – 34]	**0.012**
Pulse oximetry (%)	97 [94 – 99]	97 [95 – 99]	97 [93 – 98]	**0.003**
Body core temperature (°C)	38.1 [36.0 – 39.0]	38.0 [37.0 – 39.0]	37.0 [35.0 – 38.0]	**0.008**
Glycemia (mmol.l^−1^)	8.1 [6.1 – 10.1]	8.0 [6.1 – 10.0]	8.0 [6.1 – 10.6]	0.470
Glasgow coma scale	15 [14, 15]	15 [14, 15]	14 [12–15]	** < 10**^**–3**^
Blood lactate level (mmol.l^−1^)	4.2 ± 3.3	3.5 ± 2.9	5.7 ± 3.8	** < 10**^**–3**^
SOFA score	6 [3–9]	5 [3–8]	7 [4–10]	** < 10**^**–3**^
SAPS2 score	61 ± 22	54 ± 18	71 ± 21	** < 10**^**–3**^
Comorbidities				
Hypertension	230 (43%)	159 (44%)	71 (43%)	0.974
CHD	104 (20%)	64 (18%)	40 (24%)	0.065
Chronic cardiac failure	134 (25%)	74 (20%)	60 (37%)	** < 10**^**–3**^
CRF	151 (29%)	109 (30%)	42 (26%)	0.069
COPD	75 (14%)	45 (12%)	30 (18%)	0.144
Diabetes mellitus	151 (28%)	81 (22%)	70 (43%)	0.326
Cancer history	186 (35%)	156 (43%)	30 (18%)	**0.015**
Clinical outcomes				
In-ICU length of stay (days)	4 [2–8]	4 [2–9]	3 [1–7]	**0.007**
In-hospital length of stay (days)	10 [5–18]	13 [7–21]	5 [2–11]	** < 10**^**–3**^

*SBP* systolic blood pressure, *DBP* diastolic blood pressure, *MBP* mean blood pressure, *HR* heart rate, *RR* respiratory rate, *ICU* intensive care unit, *SOFA* sequential organ failure assessment, *SAPS2* simplified acute physiology score 2nd version, *COPD* chronic obstructive pulmonary disease, *ENT* ear nose throat, *CRF* chronic renal failure, *CHD* coronary heart disease, min = minutes. Results are expressed as mean and standard deviation for quantitative parameters (normal distribution), as median and interquartile range for quantitative parameters (non-Gaussian distribution), and as absolute value and percentage for qualitative parameters. P-value corresponds to the comparison between deceased and alive patients on day 30

Based on clinical assessment, the suspected prehospital septic shock was mainly pulmonary, digestive and urinary: 43%, 25%, and 17%, respectively. For 31 patients (6%), the prehospital septic shock origin was unknown (Table 1).

No significant difference in the duration of prehospital care was observed between survivors and deceased patients (69 ± 33 min vs. 74 ± 35 min respectively, p = 0.111; Table 1).

The average ICU LOS was 4 [2–8] days and the average in-hospital LOS was 10 [5–18] days.

Among the 530 patients cared for by a prehospital MICU and analyzed, 132 (25%) received prehospital antibiotic initiated by the MICU and 98 patients (18%) received a 3rd generation cephalosporin: 38 (7%) with cefotaxime and 60 (11%) with ceftriaxone.

Comparisons between patients with prehospital antibiotic administration and those with no prehospital antibiotic administration are summarized in Table 2. No adverse reaction related to prehospital antibiotic administration was reported among the 132 patients cared for by a prehospital mobile ICU of one of 9 French hospital centers.

**Table 2:** Comparison between patients with prehospital antibiotic administration and those with no prehospital antibiotic administration

	*No prehospital antibiotic* *(n* = *398)*	*Prehospital antibiotic* *(n* = *132)*	*P-value*
Age (years)	70 ± 15	68 ± 14	0.207
Male gender	249 (63%)	92 (70%)	0.139
Weight (kg)	74 ± 21	72 ± 17	0.254
Height (cm)	169 ± 13	171 ± 8	0.299
Initial prehospital values			
SBP (mmHg)	97 ± 31	96 ± 27	0.524
DBP (mmHg)	58 ± 20	58 ± 19	0.961
MAP (mmHg)	71 ± 23	69 ± 21	0.432
HR (beats.min^−1^)	115 ± 29	112 ± 26	0.186
RR (movements.min^−1^)	30 [23 – 36]	30 [20 – 38]	0.838
Pulse oximetry (%)	92 [85 – 96]	92 [86 – 96]	0.693
Body core temperature (°C)	38.2 [36.6 – 39.0]	38.7 [36.4 – 39.5]	0.090
Glycemia (mmol.l^−1^)	8.7 [6.0 – 12.3]	8.6 [7.0 – 11.1]	0.897
Glasgow coma scale	15 [13–15]	15 [12–15]	0.603
Blood lactate level (mmol.l^−1^)	5.8 ± 3.1	5.9 ± 4.2	0.854
Prehospital fluid expansion (ml)	868 ± 515	1102 ± 677	** < 10**^**–3**^
Norepinephrine administration	97 (24%)	58 (44%)	** < 10**^**–3**^
Norepinephrine dose (mg.h^−1^)	1.0 [0.5 – 2.0]	1.0 [0.5 – 2.0]	0.804
Prehospital duration (min)	69 ± 35	76 ± 31	**0.047**
Prehospital suspected septic shock origin			
Pulmonary	178 (45%)	52 (39%)	0.285
Digestive	99 (25%)	31 (23%)	0.748
Urinary	62 (16%)	26 (20%)	0.271
Cutaneous	25 (6%)	8 (6%)	0.928
Meningeal	5 (1%)	6 (4%)	**0.032**
Gynecological	2 (0.5%)	1 (1%)	0.737
ENT	2 (0.5%)	0 (0%)	1.000
Cardiac	2 (0.5%)	0 (0%)	1.000
Unknown	23 (6%)	8 (6%)	0.905
Final prehospital values			
SBP (mmHg)	105 ± 25	107 ± 24	0.424
DBP (mmHg)	62 ± 18	62 ± 17	0.944
MAP (mmHg)	77 ± 19	77 ± 19	0.880
HR (beats.min^−1^)	107 ± 25	104 ± 22	0.119
RR (movements.min^−1^)	25 [20–30]	24 [18–30]	0.438
Pulse oximetry (%)	97 [94 – 99]	97 [95 – 99]	0.879
Body core temperature (°C)	38.1 [36.4 – 38.8]	38.3 [36.0 – 39.1]	0.872
Glycemia (mmol.l^−1^)	8.0 [6.2 – 10.5]	8.0 [6.0 – 9.4]	0.563
Glasgow coma scale	15 [14, 15]	15 [14, 15]	0.171
Blood lactate level (mmol.l^−1^)	4.4 ± 3.4	3.8 ± 3.0	0.090
In-ICU length of stay (days)	3 [1–7]	6 [3–10]	** < 10**^**–3**^
In-hospital length of stay (days)	9 [4–17]	15 [7–21]	** < 10**^**–3**^
SOFA score	6 [3–9]	6 [3–9]	0.209
SAPS2 score	62 ± 21	56 ± 20	**0.003**
Deceased on day 30			
Comorbidities			
Hypertension	172 (43%)	58 (44%)	0.884
CHD	77 (19%)	27 (20%)	0.781
Chronic cardiac failure	109 (27%)	25 (19%)	0.054
CRF	61 (15%)	14 (11%)	0.180
COPD	52 (13%)	27 (20%)	**0.040**
Diabetes mellitus	114 (29%)	37 (28%)	0.892
Cancer history	147 (37%)	39 (30%)	0.124

*SBP* systolic blood pressure, *DBP* diastolic blood pressure, *MBP* mean blood pressure, *HR* heart rate, *RR* respiratory rate, *ICU* intensive care unit, *SOFA* sequential organ failure assessment, *SAPS2* simplified acute physiology score 2nd version, *COPD* chronic obstructive pulmonary disease, *ENT* ear nose throat, *CRF* chronic renal failure, *CHD* coronary heart disease, min = minutes.

Results are expressed as mean and standard deviation for quantitative parameters (normal distribution), as median and interquartile range for quantitative parameters (non-Gaussian distribution), and as absolute value and percentage for qualitative parameters. P-value corresponds to the comparison between patients with prehospital antibiotic administration and those with no prehospital antibiotic administration

Comparisons of 30-day mortality rate for each source for patients with prehospital antibiotic administration and those with no prehospital antibiotic administration are depicted in Table 3.

**Table 3:** 30-day mortality rates for each source for patients with prehospital antibiotics administration and those with no prehospital antibiotics administration

	No prehospital antibiotics (n = 398)		Prehospital antibiotics (n = 132)	
	Alive on day 30 (n = 269)	Deceased on day 30 (n = 129)	Alive on day 30 (n = 97)	Deceased on day 30 (n = 35)
Pulmonary	**90 (33%)**	**70 (54%)**	**54 (56%)**	**16 (46%)**
Digestive	**52 (19%)**	**32 (25%)**	**36 (37%)**	**10 (29%)**
Urinary	47 (17%)	15 (12%)	23 (24%)	3 (9%)
Cutaneous	19 (7%)	6 (5%)	7 (7%)	1 (3%)
Meningeal	5 (2%)	0 (0%)	4 (4%)	2 (6%)
Gynecological	2 (1%)	0 (0%)	1 (1%)	0 (0%)
ENT	1 (0.5%)	1 (1%)	0 (0%)	0 (0%)
Cardiac	2 (1%)	0 (0%)	0 (0%)	0 (0%)
Unknown	**18 (3%)**	**5 (12%)**	**5 (5%)**	**3 (9%)**

ENT = ear nose throat. Results are expressed as absolute value and percentage

The 30-day mortality rate was 31%: 27% (n = 35/132) among patients who received prehospital antibiotics and 32% (n = 129/398) among patients who did not receive prehospital antibiotics (p = 0.21). No patient died en route during the prehospital care.

## Fourteen patients (11%) were over diagnosed with sepsis and consequently received prehospital antibiotics unnecessarily.

### Trail target emulation

Before weighting, patients who received prehospital antibiotics differed from those who did not with respect to the following baseline characteristics: prehospital norepinephrine administration, prehospital fluid volume expansion, prehospital duration, COPD comorbidity, presumed prehospital meningeal origin of sepsis, SAPS-2 score, ICU LOS, and hospital LOS (Table 2).

**Fig. 1:**
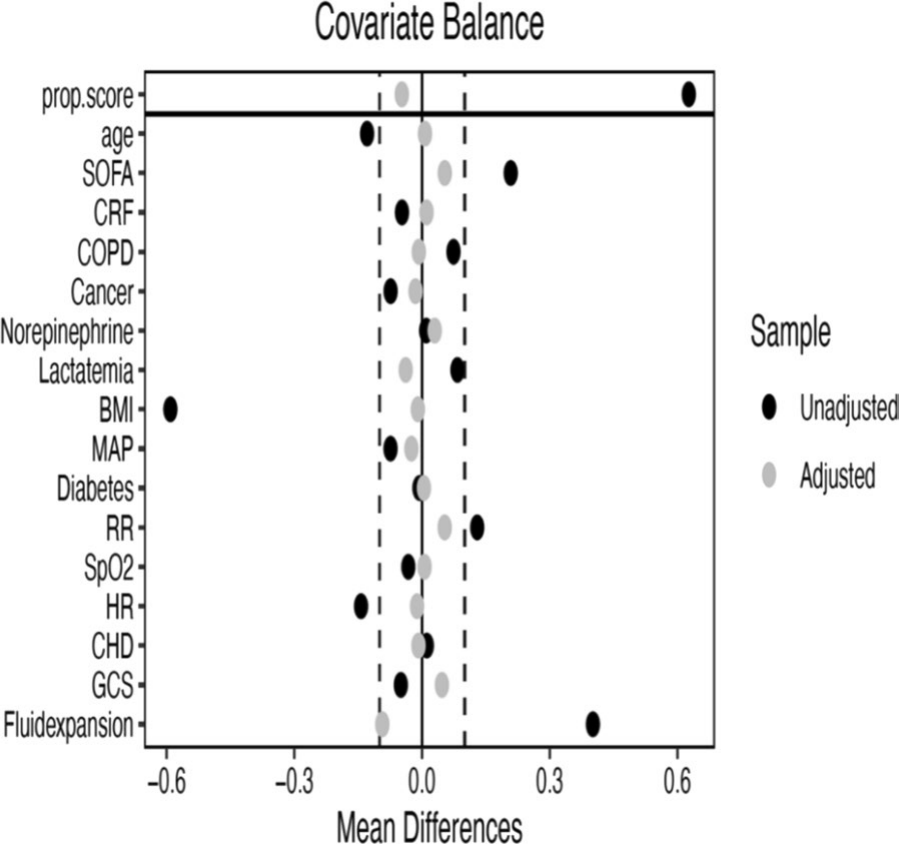
*SOFA* Sequential Organ Failure Assessment, *CRF* chronic renal failure, *COPD* chronic obstructive pulmonary disease, *BMI* body mass index, *MAP* mean arterial pressure, *RR* respiratory rate, *SpO2* pulse oximetry, *HR* heart rate, *CHD* coronary heart disease, *GCS* Glasgow coma scale

**Fig. 2:**
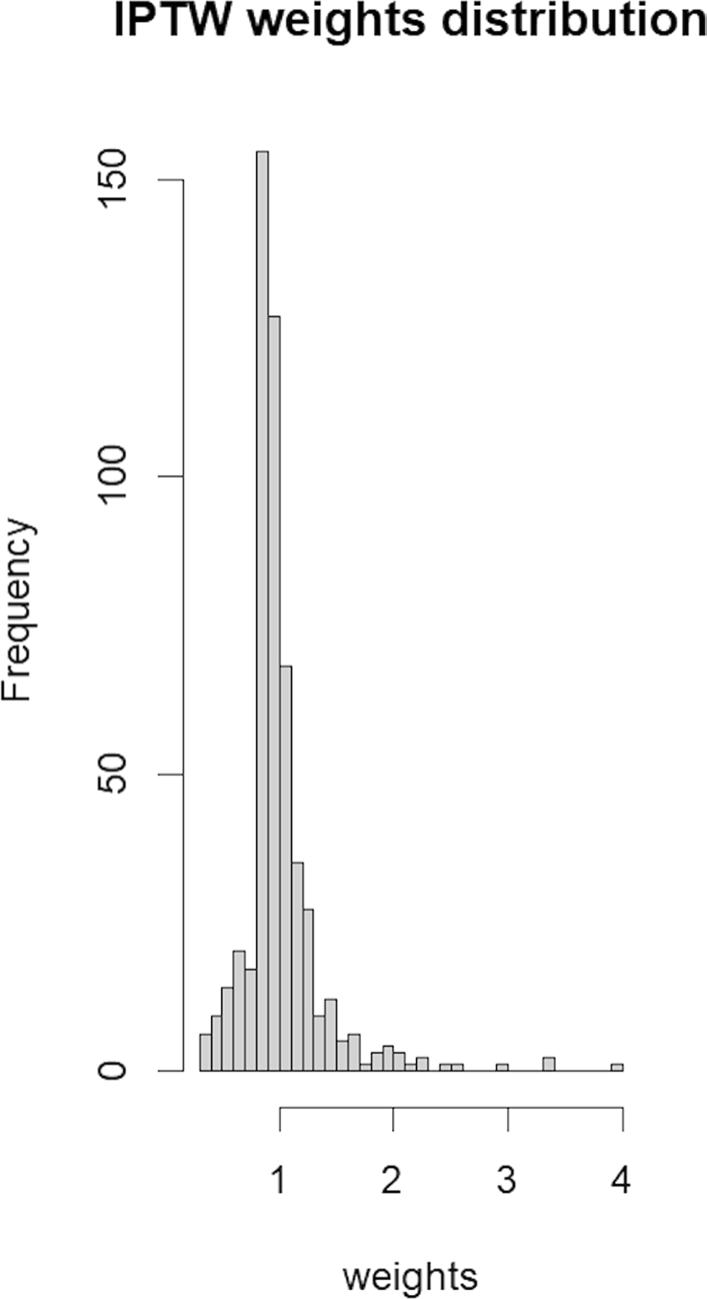
IPTW: Inverse Probability of Treatment Weighting

After application of IPTW, baseline covariates were well balanced between treatment groups, with standardized mean differences below 0.1 threshold, indicating adequate control of measured confounding (Fig. 1). IPTW weights distribution is depicted in Fig. 2.

### Primary analysis: effect of prehospital antibiotic therapy on 30-day mortality

In the IPTW-weighted analysis replicating the target trial, prehospital antibiotic administration was associated with a lower risk of 30-day mortality compared to no prehospital antibiotic administration, resulting in the effect of prehospital antibiotic administration on 30-day mortality in the ITT population with an RR = 0.64, 95%CI [0.41–0.97].

### Secondary analysis: effect of prehospital antibiotic administration depending on septic shock origin.

The weighted logistic regression model, including the same potential confounders, reported a significant association between 30-day mortality and prehospital antibiotic administration for pulmonary origin: RRa = 0.80 [0.86–0.93], urinary origin: RRa = 0.89 [0.80–0.98] and unknown origin: RRa = 0.94 [0.86–0.99] but not for cutaneous origin: RRa = 1.15 [0.97–1.33] and digestive origin: RRa = 0.96 [0.89–1.04].

## Discussion

In this study, we report that prehospital antibiotics administration was associated with a reduced 30-day mortality among septic shock patients cared for by a MICU. Prehospital antibiotics appear to be more to be more pronounced when the origin of sepsis does not require additional treatment for source control, such as in cases of pulmonary, urinary, and unknown origins.

To improve sepsis outcome, recent international sepsis guidelines updates recommend a pathway and a bundle of care including early recognition, diagnosis, and treatment [4, 8, 12]. Among treatment, antibiotics administration and hemodynamic optimization are, each and both together, associated with sepsis outcome improvement [8, 24]. For septic shock, early antibiotics administration and hemodynamic optimization have a greater treatment effect [8, 24] justifying their place in the “1-h bundle” and consequently prehospital implementation for extrahospital community septic shock [15, 18]. Septic shock mortality and morbidity are lowered by early prehospital [15] or in-hospital antibiotic therapy [25–27].

Our results are in line with the recent results of the multicenter cohort study by Lee et al. reporting the association between the prompt administration in the emergency department of appropriate antimicrobials and both a favorable prognosis and rapid defervescence, particularly among critically ill patients, regardless of the need for source control in cases of bacteremia among treatment-naïve adults with bacteremia [13]. Beyond the qualitative aspect of administering additional treatment(s) for some cases of sepsis, the delay is quantitatively important: Reitz et al. provided a comprehensive overview of the positive impact of the early source control of community-acquired sepsis on 90-day mortality [27]. Sepsis source control relies on antibiotics and sometimes one or more complementary treatments for certain causes, such as surgery for peritonitis. This may explain the reduced effectiveness of antibiotics in cases of sepsis originating in the digestive tract.

Additional treatment sometimes have a greater treatment effect and impacts more prognosis than antibiotic therapy alone [28]. Consequently, the antibiotic therapy relative weight depends on sepsis origin. This effect is even more important for the sicker patients, in other words those requiring intensive care unit admission and/or suffering from bloodstream infections [13, 29].

Our results cannot be generalized due to some limitations.

The Sepsis-3 definition is not applicable in the prehospital setting due to blood tests unavailability in the prehospital setting and the limited availability of lactate level assessments in all MICU [21]. Consequently, the Sepsis-2 definition was a more suitable and practical approach in comparison to the Sepsis-3 definition.

The study design precludes any definitive causal conclusion between 30-day mortality and prehospital antibiotics administration according to the suspected prehospital origin. Despite the use of a prespecified standardized abstraction template misclassification bias cannot be excluded. The French prehospital emergency medical services specificity based on SAMU organization and MICU practices affect the external validity. Because prehospital antibiotics administration is an empirical process, the inability to perform blood culture sampling (French MICU cannot perform blood culture) on analyzed patients may have influenced the study results and subsequent treatment strategies and patient outcomes.

The antibiotics consisted predominantly of third-generation cephalosporins, reflecting current French prehospital practice. These broad-spectrum antibiotics with incomplete coverage demonstrate efficacy against a wide range of community-acquired bacteria, encompassing those of the pulmonary, digestive, and urinary. This may limit the external validity of our findings to other healthcare systems in which antibiotic regimens are differ.

A significant proportion of patients, amounting to nearly 10%, received unnecessary prehospital antibiotics due to overdiagnosis of sepsis, with a similar a rate reported by Shappell et al. [30].

Target trial emulation based on observational data relies on the assumptions of no unmeasured confounding, positivity, consistency, and correct model specification. Caution is imperative to interpret the findings of subgroups characterized by limited sample sizes, e.g., the meningeal, gynecological, ENT, and cardiac subgroups.

Beyond these limitations, a study strength relies on the study population: patients with shock, e.g., those for whom treatment(s) initiation, especially antibiotics administration, cannot suffer from any delay. Another study strength, in the randomized controlled trial that our study aimed to replicate, admitting all patients to ICU that precluded post-randomization dropout and the associated methodological limitations.

However, the benefits and harms of early prehospital empirical antibiotics administration need to be evaluated on patient-centered outcomes (survival rates and drug side effects) and on societal outcomes (drug-resistant bacteria rates). This is an imperative consideration for caregivers prior to early administration of antibiotics.

Further prospective studies are required to confirm the prehospital antibiotics treatment effect alone or in association with others septic shock bundle of care components on septic shock outcomes.

## Conclusion

Utilizing a target trial emulation framework, we ascertained that the initiation of prehospital antibiotics administration was associated with a reduced 30-day mortality among septic shock patients cared for by a MICU. By aligning treatment assignment, time zero, and follow-up with a hypothetical randomized trial, our analysis strengthens causal interpretation compared with conventional observational approaches. Nevertheless, residual confounding factors may remain; thus, our results need to be confirmed by randomized or pragmatic trials.

## Data Availability

Data will be made available on reasonable request.

## Abbreviations

CCF: Chronic cardiac failure
CHD: Coronary heart disease
COPD: Chronic obstructive pulmonary disease
CRF: Chronic renal failure
DAP: Diastolic arterial pressure
GCS: Glasgow coma scale
HR: Heart rate
ICU: Intensive care unit
IPTW: Inverse probability treatment weighting
LOS: Length of stay
MAP: Mean arterial pressure
RR: Respiratory rate
RRa: Risk ratio adjusted
SAMU: Urgent medical aid service
SAP: Systolic arterial pressure
SMUR: Mobile emergency and resuscitation service
SAPS-2: Simplified acute physiology score
SOFA: Sequential organ failure assessment
SpO2: Pulse oximetry
